# Non-Melanoma Skin Cancer: Dermatoscopic Diagnostic Clues in Mexican Individuals Based on Fitzpatrick Skin Phototypes

**DOI:** 10.3390/jcm14092966

**Published:** 2025-04-25

**Authors:** Esli Camila Sánchez Moreno, Andrea Carolina Machado Sulbaran, Lizbeth Riera Leal, Yveth Marlene Ortiz García, Luis Roberto Olivas Román, Annie Riera Leal

**Affiliations:** 1 Dermatology Department, General Hospital of the State of Sonora, Hermosillo 83000, Mexico; eslicamila@gmail.com; 2 Research and Higher Education Center of UNEPROP, Hermosillo 83105, Mexico; rieraleal@yahoo.com.mx (L.R.L.); yveth.ortiz@academicos.udg.mx (Y.M.O.G.); 3 Childhood and Adolescence Cancer Research Institute, University Center of Health Sciences, University of Guadalajara, Guadalajara 44100, Mexico; andrea.machado5223@academicos.udg.mx; 4 Dermatology Department, Ayala Hospital-HGR 45 IMSS, Guadalajara 44100, Mexico; 5 Research Institute of Dentistry, University Center of Health Sciences, University of Guadalajara, Guadalajara 44100, Mexico; 6 Pathology Department, General Hospital of the State of Sonora, Hermosillo 83000, Mexico; luis_chaparro@hotmail.com

**Keywords:** non-melanoma skin cancer, dermatoscopy, melanin pigment, and vessel arrangements and patterns

## Abstract

**Background/Objectives**: Skin cancer is increasingly prevalent. Non-melanoma skin cancers pose a challenge, as most lesions are diagnosed at later stages and often lead to complications. Although dermatoscopy has emerged as a valuable tool that enhances the confidence of dermatologists, specific patterns for accurately identifying various subtypes of non-melanoma skin cancer have yet to be detailed. This study aimed to investigate dermatoscopic clues that facilitate accurate diagnosis of non-melanoma skin cancer among Mexican individuals. There is insufficient acknowledgment of high skin cancer rates among non-Whites. **Methods**: The study included fifty-three patients diagnosed with non-melanoma skin cancer, aged 39 to 89, who visited an academic dermatology department for skin examinations. Two certified dermatologists evaluated at least three dermatoscopy images for each lesion. A biopsy was taken to confirm the preliminary diagnosis. Statistical analysis was performed using GraphPad Prism v8.0, considering a probability (*p*) value of less than 0.05 as significant. **Results**: Most patients were classified as phototype III. Patients with phototype IV were younger at the time of diagnosis. Basal cell carcinomas were the most common cancer subtype. Nodular and ulcerated tumors were the most prevalent morphology. The dermatoscopic examination revealed that 60% of the lesions were pigmented, with a predominance of polymorphic vascular patterns. Squamous cell carcinomas exhibited monomorphic vascular structures. Both groups’ blood vessel arrangements and specific patterns were primarily radial. **Conclusions**: Phototypes III and IV are predominant in the Mexican population; however, patients with non-melanoma skin cancer tend to be under 60 years of age at diagnosis. Although prominent reticular lines were distinctive of the ulcerated lesions, finding any pathognomonic pigmentary feature for non-melanoma skin cancer subtypes or locations was impossible. A polymorphic pattern of blood vessels, with a predominance of linear vessels, typically indicates the presence of Basal cell carcinoma. In contrast, a monomorphic pattern with a predominance of comma vessels is more suggestive of Squamous cell carcinoma.

## 1. Introduction

Skin cancer presents a life-threatening health issue if left untreated [1,2]. Although the skin is the most visible organ, many lesions can easily be misidentified as skin cancer. It is particularly concerning that skin cancer is often underdiagnosed until it progresses to advanced stages, as clinical characteristics are not always sufficient to identify a definitive signature of the disease. It can be categorized into two groups: non-melanoma skin cancer (NMSC) and melanoma. Among the former, 95% of cases are basal cell carcinoma (BCC) or squamous cell carcinoma (SCC), but other rare malignant skin tumors also fall under the NMSC classification [1,3].

Research from various electronic databases indicates that the types and subtypes of skin cancer are becoming increasingly common among clinically diagnosed cancers worldwide [4]. Despite darker-skinned patients being affected at lower rates compared to their lighter-skinned counterparts, this group reports higher mortality rates. Biological differences, such as genomic and melanin-related variations [5,6], along with disparities in healthcare utilization, significantly contribute to these outcomes. Melanin pigmentation protects the skin from the harmful effects of UV radiation, serves various functions, and has diverse structures and forms. Pigmentary traits are genetically determined and exhibit a polygenic inheritance pattern [7]. In dermatology practice, the structure and functional behavior of the skin are influenced by phototypes, and environmental factors also play an essential role [8]. Ancestral background is critical because natural selection shapes the overall genetic architecture of skin pigmentation [9].

Dermatoscopy is a non-invasive, in vivo technique that adds a new dimension to evaluating subsurface skin structures in the epidermis, dermo-epidermal junction, and upper dermis [10]. The correlation between dermatoscopic and histopathological findings enhances the quality of skin cancer diagnoses and boosts dermatologists’ confidence. Dermatoscopy operates parallel to the skin surface, analyzing structures on a horizontal plane, while pathology examines sections on a vertical plane. The color of dermatoscopic features varies based on the histopathological levels within the epidermis and superficial dermis. Pigmented structures appear black in the cornified layer, brown at the dermo-epidermal junction, and gray-blue in the papillary dermis [10]. Dermatoscopic–pathologic correlation has revealed additional features such as brown globules, hypopigmented areas, white regions, and whitish veils that correlate well with melanocytic lesions [11]. The situation is less clear for non-melanocytic lesions.

Visualizing and identifying vessels with a characteristic morphology can be the key to diagnosis, especially in NMSC, where vascular features are often the only clues. A significant body of evidence highlights the importance of aberrant angiogenesis in cancer pathogenesis. Growing tumors feed on newly formed capillaries. Identifying and evaluating vascular structures on dermatoscopy depends mainly on the optical system and examination technique. The method (contact dermatoscopy vs. polarized light dermatoscopy), the dermatoscope resolution, and the choice of immersion fluid are considered [12]. Thanks to its high viscosity, a range of immersion fluids can be used, making the ultrasound gel the favorite for some authors when examining vessels without polarized light. A magnification of at least 30× is recommended for visualizing tiny capillaries [13]. The most crucial chromophore in non-pigmented cutaneous tumors is hemoglobin.

Some vascular features are small and usually occluded by other structures, making their detection challenging. The predominant vascular pattern will also depend on the depth of lesions, the volume of the tumor, and its proliferation pattern. Tumor topography and morphology, phototype, and age are essential clinical aspects. The vessel morphology, architectural arrangement, and additional defects should be analyzed. Several studies have sought to identify vascular features associated with the more aggressive NMSC phenotypes or initial, subtle lesions.

Diagnosing NMSC solely through clinical and dermatoscopic findings can be challenging in individuals with darker skin tones. Additionally, various benign and malignant lesions can present differential diagnoses. For instance, the significant variety in dermoscopy of melanin pigmentation patterns means that nevi and melanoma often mimic BCC [14]. Conversely, lesions such as actinic keratosis and keratoacanthoma, common in lighter skin due to chronic photodamage, are less prevalent among individuals with darker skin tones. In other studies, specific dermoscopic criteria for sebaceous keratosis, dermatofibroma, angiomas, and sebaceous hyperplasia are infrequent or absent in individuals with darker skin tones [14,15].

We undertook this work to determine whether the melanin and vascular patterns observed in dermatoscopy might reflect the biological behavior of NMSC in a Mexican population. Given the large Indigenous populations, ethnicity in Mexico is regarded as a “risk factor” for several diseases, warranting special attention and concern. The paternal ancestry estimated in western Mexico was predominantly European, followed by Amerindian and African (approximately 60%, 25%, and 15%) [9]. Significant genetic heterogeneity was established. Analyzing pigment and vascular morphological and distribution patterns in Mexican patients with NMSC can further support diagnosis, assessment, and monitoring.

## 2. Methodology

### 2.1. Selection of Patients

We conducted an institutionally approved transversal study that included patients diagnosed with NMSC in an academic dermatology setting (Dermatology Department in the Sonora State General Hospital) from April 2024 to July 2024. Two independent reviewers obtained data from Electronic Medical Records (EMR). The prevalence of NMSC in the general adult population was taken as a reference, where it is reported to be around 3%. The equation used was n = Z^2^ × P × 1 − P∕d^2^. Participants self-assessed their skin types using the Fitzpatrick Skin Phototype Classification questionnaire, which employs a six-point categorical scale. The FSPC is the most widely utilized tool for evaluating skin phototypes based on the amount of pigment in the skin and its reactions to sun exposure, with questions such as the following: “What is the color of your skin?”, “What is your hair’s natural color?”, “What is the color of your eyes?”, “Do you have freckles on unexposed areas?”, “Do you turn brown within several hours of sun exposure?”, and “To what degree do you turn brown?”. Scores and corresponding skin phototypes: 0–7: I, 8–16: II, 17–25: III, 26–30: IV.

Patients with a clinical suspicion were evaluated under dermatoscopy using a Dermlite DL5 dermatoscope (DermLite LLC, San Juan Capistrano, CA, USA). At least three images were captured under PD and UV light (365 nm). The images were obtained using an iPhone 15 Pro Max (model MU683LL/A) and were stored in the database until analysis. A total of fifty-three patients with NMSC were identified. Two expert dermatologists independently analyzed the images. Then, a 5 mm punch biopsy was taken to confirm the preliminary diagnosis in each patient. A positive clinicopathological correlation for any NMSC underwent excisional surgery and defect repair by direct closure, flap, or graft. Two certified histopathologists performed a histopathological analysis of the entire sample. This study was conducted according to the Helsinki Declaration. It was approved by “Comité Ética en Investigación CONBIOÉTICA 26-CEI-00220170517” at the Sonora State General Hospital. Protocol code: CEI-2024-8, 13 June 2024. Informed written consent was obtained from all subjects to participate in the research at every step and to publish images.

### 2.2. Statistical Analysis

Statistical analysis was performed using GraphPad Prism v8.0. A probability (*p*) value of less than 0.05 was considered significant. Categorical variables were expressed as percentages and counts. The Shapiro–Wilk normality test was applied to verify the distribution of the data. Continuous variables are expressed as mean and standard deviation (parametric distribution) or median with an interquartile range (nonparametric distribution), depending on the distribution. Differences in frequencies were compared using the Chi-square (*p*) or Fisher’s exact test (*p*′). The Mann–Whitney U test was used to evaluate differences between two groups, whereas the Kruskal–Wallis test was used to analyze three or more groups. Spearman test was used to assess correlations between quantitative data.

## 3. Results

**Figure 1:**
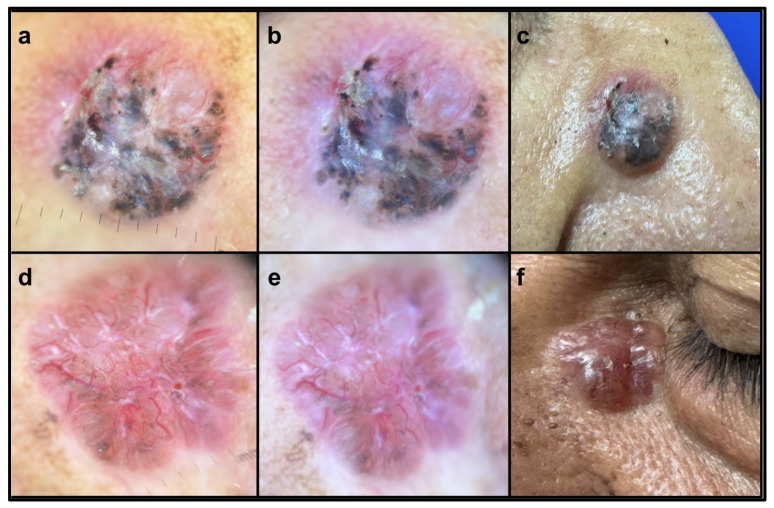
Non-polarized dermatoscopy (**a**,**d**): superficial details, such as white areas and pigment, are more pronounced. Polarized dermatoscopy (**b**,**e**): better visualization of the arborizing vessels and deeper structures are provided. (**c**,**f**) depict the clinical characteristics of the lesions.

The average age of the patients was 69 years, with the majority classified as phototype III (60.4%). Tumors had an average duration of 2.39 years. Most were asymptomatic (52.8%) or presented with pruritus (30.2%) or pain (17%), primarily in the nodular or ulcerated forms (Table 1). No correlations were found between age and clinical variables such as duration of lesions (years), lesion size (cm), or depth of invasion (mm). A positive correlation was identified between lesion duration (years) and lesion size (cm) (*p* < 0.0001, r = 0.6934, 95% confidence interval 0.514–0.814). Figure 1 presents two clinical examples.

Women, on average, were older (72 ± 14.8) than men (67 ± 13.5), with no statistical differences (*p* = 0.1). However, a trend in the data indicated a longer tumor duration in men (2.05 ± 1.12 years versus 2.69 ± 1.83 years, *p* = 0.1), as well as larger lesion sizes: 1.99 ± 1.66 cm in women compared to 3.59 ± 3.55 cm in men (*p* = 0.09). In general, patients with ≥ 3 years of evolution had larger lesions (4.82 ± 3.53) than those with 1–2 years (1.49 ± 1.23) (*p* < 0.0001).

**Figure 2:**
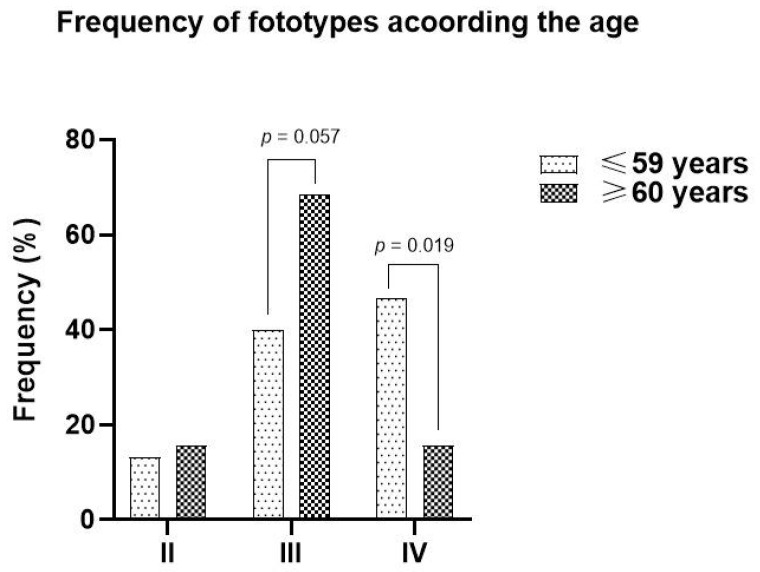
Distribution of patients according to the phototypes and age of non-melanoma skin cancer diagnosis.

Interestingly, patients with darker phototypes were younger at diagnosis (Figure 2), suggesting potential underlying factors in the Mexican population that may promote skin cancer development independent of UV radiation exposure.

**Figure 3:**
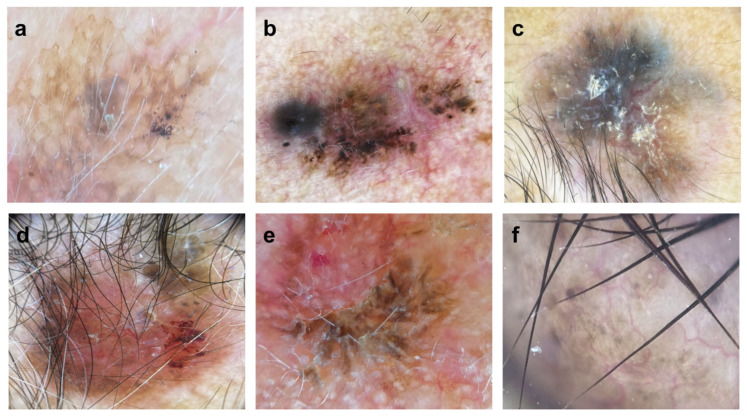
Dermatoscopic images displaying melanin pigment structures. (**a**): reticular light brown lesions. (**b**): dark brown pigment globules, accompanied by black pigment and a gray veil at the edges. (**c**): blue-black pigmentation with patches of light brown and structureless patterns. (**d**): structureless brown pigmentation at the edges, along with unstructured blue-black-gray pigmentation. (**e**): brown pigmentation at the edges, featuring angled lines. (**f**): dark brown dots.

BCC represented the most prevalent type of NMSC, comprising 81.1%. SCC accounted for 16.98%, while Dermatofibrosarcoma Protuberans comprised 1.89% of the diagnosed cases. Pigmentation was noted in 60.5% of BCC patients (*p* = 0.001), while keratin scales were observed in 77.8% of SCC patients (*p* < 0.0001) (Table 2). Gray or blue coloration, milium-type cysts, and pseudo-follicular openings were exclusively reported in patients with BCC, without statistical differences (Figure 3).

Analyzing the topography, BCC was most commonly found on the nose (27.9%), cheek (25.6%), and forehead (11.6%), with no statistical difference. In contrast, SCC was most frequently located on the lips (33.3%, *p* < 0.0001), chin (11.1%, *p* = 0.027), and thumb (11.1%, *p* = 0.027). Patients with ulcerated lesions experienced a longer duration of lesions and larger lesion sizes (2.89 ± 1.87 years and 1.84 ± 0.89 cm) compared to those without ulcers (1.84 ± 0.89 years and 2.07 ± 1.91 cm; *p* = 0.014 and *p* = 0.058, respectively). Patients with nodular lesions were younger (66 ± 14.6 years) and had smaller lesion sizes (2.25 ± 1.95 cm) than those with non-nodular lesions (74.5 ± 12.1 years and 3.81 ± 3.88 cm), though no statistically significant differences were noted.

**Figure 4:**
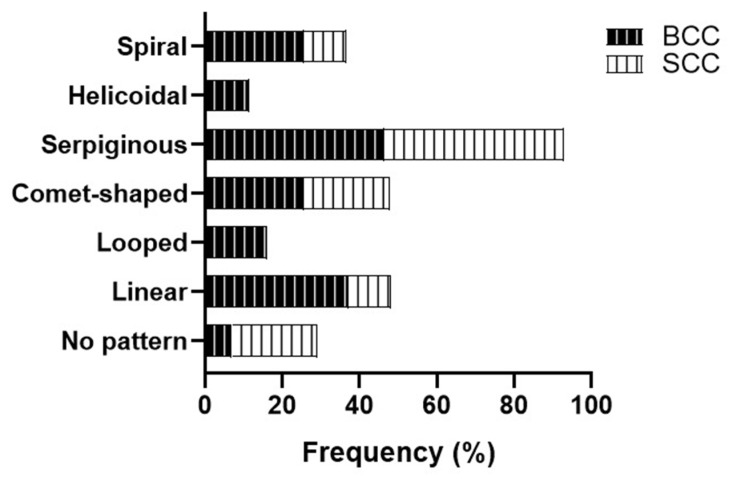
Vascular pattern according to the type of non-melanoma skin cancer. BCC: basal cell carcinoma; SCC: squamous cell carcinoma.

Serpiginous vessels were the most common vascular pattern found in both BCC and SCC (46.5%). In BCC, the second most prevalent pattern was the linear pattern (37.2%), whereas in SCC, the comet morphology of vessels was the second most prevalent pattern (22.2%). Patients lacking a specific vascular pattern were those with SCC (22.2%), showing no significant statistical differences (Figure 4).

**Figure 5:**
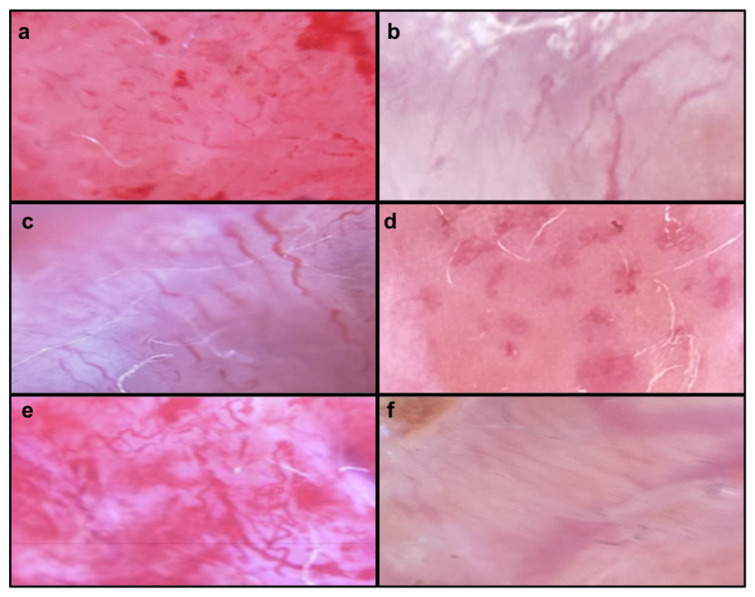
Dermatoscopic images displaying vessel structures. (**a**): curved. (**b**): looped. (**c**): serpentine. (**d**): coiled. (**e**): helical. (**f**): straight.

BCC neoplasms exhibit a higher frequency of polymorphic vessel patterns (67.5%), while those with SCC tend to present a monomorphic pattern (55.6%). Most patients with BCC showed serpiginous (44.2%) and linear (37.2%) structures within the linear blood vessel subtype. Meanwhile, many SCC patients did not exhibit a specific subtype (33.3%, *p* = 0.055). Both groups’ blood vessel arrangements and specific patterns were primarily radial (65.1% in BCC and 44.4% in SCC) (Figure 5).

Ulcerated tumors were common in our sample (52.8% of patients). In 82.1% of cases, they displayed prominent reticular lines associated with perineural invasion in the histopathology images from 10.7% of patients. These characteristics were found more frequently in ulcerated than non-ulcerated lesions (*p* = 0.009 and *p* = 0.09, respectively). In contrast, non-ulcerated lesions had a higher frequency of peripheral black lumps, 24.0% (*p* = 0.009), in addition to milium-like cysts, pseudo-follicular openings (*p* = 0.09), and pigmentation (*p* = 0.003) (Table 3).

Furthermore, 60.4% of the tumors had nodular morphology, which showed a higher frequency of the linear helical blood vessel subtype (*p* = 0.035). Patients with nodular lesions more frequently had ulcerated surfaces than those with non-nodular lesions, which had rough surfaces (*p* = 0.0038, *p* = 0.009). Also, in non-nodular lesions, it was more common to see a lack of specific blood vessel arrangement patterns (*p* = 0.06) and keratinization (*p* = 0.0008) (Table 4).

## 4. Discussion

Despite the lack of recognition of high skin cancer rates among non-Whites, our increasing incidence underscores the need for enhanced preventive and diagnostic programs. The rising life expectancy of the general population is likely contributing to the prevalence of dermatological NMSC in elderly patients. However, a significant portion of our study population was under 60. Notably, the younger demographic exhibited higher phototypes. These findings emphasize the need to consider additional factors beyond UV radiation as risks for skin cancer.

A comprehensive genome-wide association study (GWAS) and Polygenic Risk Score (PRS) analysis of 13 pigmentary-related traits was conducted to assess phototype as a genetic proxy for skin functionality and disease in open mixed populations [16]. This study revealed a strong link between fair phototypes and NMSC, OR = 0.93; BCC, OR = 0.97). Abnormal melanin physiology has been associated with the immunomodulation of the tumor microenvironment [17]. Moreover, variations in sun-protective behaviors can be observed among sun-sensitive individuals based on race and ethnicity [8]. A history of severe sunburns has been connected to both BCC and SCC [18].

BCC was the most common form of NMSC, with the nodular subtype being the most prevalent. A study recently implicated incipient ulceration in cutaneous melanomas as a significant prognostic factor for survival rates [19]. In our sample, most patients presented with ulceration at diagnosis. Clinical interpretive difficulties may arise when a NMSC exhibits incipient ulceration; furthermore, we did not find a study that described a significant impact of ulceration on survival in this type of tumors. The biological mechanism of ulceration is likely multifactorial, although the tumor growth rate appears to be a determining factor [20]. The question arises whether, in the Mexican population, despite BCC being universally regarded as a tumor of low malignancy, ulceration could act as a surrogate marker for aggressive tumor biology.

Pigmented lesions of BCC were predominant. However, the pattern of lesions displaying melanin and BCC-related pigmented structures, such as large gray-blue ovoid nests, multiple gray-blue globules, maple leaf-like areas, and spoke-wheel areas [11,21], did not dominate the dermatoscopic features. A less commonly recognized dermatoscopic feature, the blue-white veil, was observed in some of our patients and was significantly associated with non-ulcerated BCC. In one study, short fine telangiectasias, leaf-like areas, spoke-wheel areas, small erosions, and concentric structures were significantly linked only to BCC’s superficial variant [22]

Other rare findings, such as milium-type cysts and pseudo-follicular openings, were exclusively noted in patients with BCC, showing no statistical differences. Among the six positive characteristics of Menzies’ algorithm (arborizing telangiectasias, ulceration, blue-gray ovoid nests, blue-gray globules, leaf-like areas, and spoke-wheel areas) [11], the presence of ulcers was significantly associated, particularly in patients with a longer duration of lesions at the time of diagnosis.

The distribution of specific NMSC-associated pigmentary patterns did not differ between BCCs and SCCs. Aside from the presence of melanin-dotted pigment and thick reticular lines, both tumors exhibited roughly the same associations with individual dermatoscopic criteria. Keratin scales were the most common dermatoscopic findings in SCC. It has been noted that keratin/scales, blood spots, white circles, and structureless areas are typically present in SCC [23]. Benati et al. reported that scales, white structureless areas, and white halos were observed in most cases (100%, 91%, and 86%, respectively) [24].

Despite heavily pigmented structures, we observed a high occurrence of vascular patterns. An inverse relationship has been reported between the degree of pigmentation in BCC and the presence of any vascular structure [22]. At least one vascular pattern was noted in our patients’ lesions. In contrast to the work of Arpaia et al., in which the arborizing pattern in the ulcerated portion was associated with a correct diagnosis [25], our sample did not demonstrate this vascular pattern. The polymorphic vessels were described only in the BCC. In some studies, the presence of vessels covering more than half of the tumor surface, along with diffuse distribution and bleeding, significantly increased the likelihood of SCC [23]. A monomorphic pattern characterized by a predominance of comma vessels was more prevalent in our patients.

## 5. Conclusions

Several research groups have studied the use of dermatoscopy to enhance the detection and differentiation of skin cancer. However, aside from melanoma, there are no well-defined algorithms for distinguishing between the specific types of NMSC, particularly in patients with skin phototypes III and IV. In our study, phototype III predominated among patients diagnosed with NMSC, who were also younger at the time of diagnosis (under 60 years) compared to what is reported in the literature. This highlights the need to consider, in addition to UV radiation, other risk factors associated with NMSC development in the Mexican population. As the duration of the pathology increases, the lesions evolve from a nodular to an ulcerated nodular morphology. Consequently, ulceration in our patients was significantly associated with larger size and prolonged duration. From a dermatoscopic perspective, the ulcerated lesions displayed prominent reticular lines.

The patterns associated with pigment alterations generally did not demonstrate sufficient effectiveness to support the use of dermatoscopy in differentiating between types of NMSC. NMSC lesions in the Mexican population displayed various blood vessel types and patterns. Serpentine vessels predominated in both main groups (BCC and SCC). However, our results indicate that a polymorphic pattern with a predominance of linear vessels suggests a diagnosis of BCC. In contrast, a monomorphic pattern with a predominance of comma vessels is more closely associated with a diagnosis of SCC. The classically described dermatoscopic findings to identify BCC did not prevail in our sample despite this type of NMSC being the most diagnosed. This could be related to the phototype and genetic factors, among others in the Mexican population.

### Limitations

This study’s limitations include its cross-sectional nature. Further controlled studies are necessary to confirm our preliminary results.

## Figures and Tables

**Table 1:** Clinical and demographic data.

	Total % (n = 53)
Patient age (years)	69.37 ± 14.18
Sex	
Female	45.3 (24)
Male	54.7 (29)
Skin phototype	
II	15.1 (8)
III	60.4 (32)
IV	24.5 (13)
Sun exposure history	100 (53)
Use of sun protection	0
Duration of lesion (years)	2.39 ± 1.57
Symptoms	
Asymptomatic	52.8 (28)
Pruritus	30.2 (16)
Pain	17.0 (9)
Morphology type	
Nodular	26.6 (14)
Ulcerated nodular	5.7 (3)
Nodular with telangiectasias	1.9 (1)
Papule	5.7 (3)
Pigmented papule	1.9 (1)
Patch shape	11.3 (6)
Warty appearance	1.9 (1)
Exulceration	41.5 (22)
Neoformation	1.9 (1)
Exophytic tumor mass	1.9 (1)
Ulcerated lesions	32.1 (17)
Non-ulcerated lesions	56.6 (30)
Partially ulcerated lesions	11.3 (6)

**Table 2:** Pattern of lesions pigmented by melanin according to the type of non-melanoma skin cancer. BCC: Basal cell carcinoma; SCC Squamous cell carcinoma. Differences in frequencies were compared using the Chi-square (*p*)/Fisher’s exact test (*p*′). * *p* = 0.001/*p* = 0.002; ** *p* = 0.0029/*p* = 0.0047.

Pattern Analysis of Lesions Pigmented by Melanin	BCC % (n = 43)	SCC % (n = 9)	Ulcerate % (n = 28)	Non-Ulcerate % (n = 25)	Nodular % (n = 32)	Non-Nodular % (n = 21)
Pigmented lesion	60.5 (26) *	0	50.0 (14)	48.0 (12)	65.6 (21) **	23.8 (5)
Monomorphic	30.2 (13)	55.6 (5)	28.6 (8)	40.0 (10)	31.3 (10)	38.1 (8)
Polymorphic	67.4 (29)	44.4 (4)	71.4 (20)	56.0 (14)	65.6 (21)	61.9 (13)
Pigmented areas	32.6 (14)	33.3 (3)	32.1 (9)	32.0 (8)	46.9 (15)	28.6 (6)
Clods (includes dots)	7.0 (3)	0	3.6 (1)	8.0 (2)	9.4 (3)	0
Depigmented areas	14.0 (6)	0	10.7 (3)	12.0 (3)	6.3 (2)	19.0 (4)
White structures	65.1 (28)	66.7 (6)	57.1 (16)	72.0 (18)	59.4 (19)	71.4 (15)

**Table 3:** Dermatoscopic findings are determined by the type of non-melanoma skin cancer and the presence of ulceration: clinical variables, pigment structures, and patterns. BCC: basal cell carcinoma; SCC: squamous cell carcinoma. Frequencies were compared using the Chi-square test (*p*) or Fisher’s exact test (*p*′). *p^a^* indicates the comparison between types of ulcerated non-melanoma skin cancer (BCC and SCC); *p^b^* indicates the comparison between ulcerated and non-ulcerated lesions of SCC; *p^c^* indicates the comparison between ulcerated and non-ulcerated lesions of BCC; *p^d^* indicates the comparison between types of non-ulcerated non-melanoma skin cancer (BCC and SCC).

	Ulcerated Tumors % (n = 28)		Non-Ulcerated Tumors % (n = 25)		*p^a^*	*p^a^*′	*p^b^*	*p^b^*′	*p^c^*	*p^c^*′	*p^d^*	*p^d^*′
Variables	BCC % (n = 24)	SCC % (n = 3)	BCC % (n = 19)	SCC % (n = 6)								
**Symptoms Associated**												
Asymptomatic	58.3 (14)	33.3 (1)	57.9 (11)	33.3 (2)								
Pruritus	25.0 (6)	33.3 (1)	36.8 (7)	16.7 (1)								
Pain	16.7 (4)	33.3 (1)	5.3 (1)	50.0 (3)							0.009	0.03
**Edges of the lesion**												
Well defined	12.5 (3)	0	52.6 (10)	16.7 (1)	0.004	0.007						
Irregular	37.5 (9)	0	15.8 (3)	16.7 (1)	0.051							
Poorly defined	37.5 (9)	100 (3)	26.3 (5)	66.7 (4)							0.073	
**Melanin pigment**												
Monomorphous	25.0 (6)	66.7 (2)	36.8 (7)	50.0 (3)								
Polymorphous	75.0 (18)	33.3 (1)	57.9 (11)	50.0 (3)								
Pigmented areas	37.5 (9)	66.7 (2)	42.1 (8)	33.3 (2)								
Gray or blue color	8.3 (2)	0	10.5 (2)	0								
Dotted pigment	79.2 (19)	0	42.1 (8)	83.3 (5)	0.013	0.025	0.017	0.048	0.004	0.019		
Peripheral black lumps	8.3 (2)	0	26.3 (5)	16.7 (1)								
Clods (includes dots)	4.2 (1)	0	10.5 (2)	0								
Depigmented areas	12.5 (3)	0	15.8 (3)	0								
**Types of lines**												
Thick reticular lines	83.3 (20)	66.7 (2)	36.8 (7)	83.3 (5)	0.002	0.004					0.047	0.07
Segmental radial lines	25.0 (6)	33.3 (1)	15.8 (3)	16.7 (1)								
white lines	58.3 (14)	66.7 (2)	66.7 (13)	66.7 (4)								
Angulated lines	0	0	0	0								
Lines parallel	0	0	0	0								
**Melanin pigment distribution**												
Central	16.7 (4)	0	21.1 (4)	0								
Peripheral	12.5 (3)	66.7 (2)	21.1 (4)	33.3 (2)								
Peripheral and central	8.3 (2)	0	10.5 (2)	0								
Not applicable	62.5 (15)	33.3 (1)	47.4 (9)	66.7 (4)								
**White structures presence**	54.2 (13)	100 (3)	78.9 (15)	50.0 (3)	0.09							
**Pattern of white structures**												
Amorphous	25.0 (6)	0	26.3 (5)	33.3 (2)								
Irregular	25.0 (5)	66.7 (2)	26.3 (5)	16.7 (1)								
Dotted	4.2 (1)	0	5.3 (1)	0								
Reticulated	0	0	10.5 (2)	0								
Linear	0	33.3 (1)	0	0								
Homogeneous	0	0	5.3 (1)	0								
Radial	0	0	5.3 (1)	0								
Disrupted	8.3 (2)	0	0	0								

**Table 4:** Dermatoscopic findings are determined by the type of non-melanoma skin cancer and the presence of ulceration: vessel structure, arrangement, and specific patterns. BCC (basal cell carcinoma) and SCC (squamous cell carcinoma). Differences in frequencies were analyzed using either the Chi-square test (*p*) or Fisher’s exact test (*p*′). “*p^a^*” represents the comparison between ulcerated non-melanoma skin cancers (BCC and SCC); “*p^b^*” indicates the comparison between ulcerated and non-ulcerated SCC lesions; “*p^c^*” denotes the comparison between ulcerated and non-ulcerated BCC lesions; “*p^d^*” represents the comparison between non-ulcerated non-melanoma skin cancers (BCC and SCC).

	Ulcerated Tumors % (n = 28)		Non-Ulcerated Tumors % (n = 25*)*		*p^a^*	*p^a^*′	*p^b^*	*p^b^*′	*p^c^*	*p^c^*′	*p^d^*	*p^d^*′
Vascular Variables	BCC % (n = 24)	SCC % (n = 3)	BCC % (n = 19)	SCC % (n = 6)								
**Vessel Structures**												
No structure	25.0 (6)	66.7 (2)	47.4 (9)	50.0 (3)								
Dott	66.7 (16)	0	47.4 (9)	83.3 (5)			0.018	0.048	0.027	0.057		
Clod	4.2 (1)	0	5.3 (1)	0								
Linear	25.0 (6)	66.7 (2)	42.1 (8)	50.0 (3)								
**Linear vessel subtypes**												
No pattern	8.3 (2)	0	10.5 (2)	50.0 (3)								
Linear straight	33.3 (8)	0	42.1 (8)	16.7 (1)								
Linear looped	12.5 (3)	0	21.1 (4)	0								
Linear curved	29.2 (7)	33.3 (1)	21.1 (4)	16.7 (1)								
Linear serpentine	54.2 (13)	66.7 (2)	31.6 (6)	33.3 (2)								
Linear helical	12.5 (3)	0	15.8 (3)	0								
Linear coiled	25.0 (6)	33.3 (1)	26.3 (5)	0								
**Arrangement of vessels and specific patterns**												
Random	8.3 (2)	33.3 (1)	21.1 (4)	0								
Clustered	0	0	0	0								
Serpinginous	0	0	0	0								
Radial	70.8 (17)	33.3 (1)	57.9 (11)	50.0 (3)								
Reticular	0	0	0	0								
Branched	0	0	5.3 (1)	0								
**Other variables**												
Maple Leaf Structure	0	0	0	0								
Radiated Areas	37.5 (9)	66.7 (2)	21.1 (4)	16.7 (1)								
Blue-Gray Ovoid Nests	33.3 (8)	33.3 (1)	42.1 (8)	50.0 (3)								
Unstructured eccentric area	0	33.3 (1)	5.3 (1)	0								
Millium pseudocysts and follicular pseudoapertures	8.3 (2)	0	10.5 (2)	0								
Pigmented Reticulum, Aggregations of Black Dots and Whitish Veil	12.5 (3)	66.7 (2)	26.3 (5)	0			0.023	0.083	0.023	0.079		

## Data Availability

The original contributions presented in this study are included in the article. Further inquiries can be directed to the corresponding author(s).
