# Expression of Concern: Sánchez Moreno et al. Non-Melanoma Skin Cancer: Dermatoscopic Diagnostic Clues in Mexican Individuals Based on Fitzpatrick Skin Phototypes. *J. Clin. Med.* 2025, *14*, 2966

**DOI:** 10.3390/jcm15114348

**Published:** 2026-06-04

**Authors:** Journal of Clinical Medicine Office

**Affiliations:** MDPI, Grosspeteranlage 5, 4052 Basel, Switzerland; miriam.havrisciuc@mdpi.com

With this notice, the *Journal of Clinical Medicine* Editorial Office wishes to alert readers to concerns related to this article [1]. Following publication, concerns were raised regarding authorship, data ownership, and the ethical approval of the reported research involving human participants. We have been informed that these matters are currently the subject of ongoing legal proceedings. An investigation is being conducted by the Editorial Office in consultation with the Editor-in-Chief. As this process is ongoing, the journal is not in a position to reach a conclusion at this time. The Editorial Office will provide an update once the investigation and related proceedings have been concluded and will implement any post-publication actions deemed necessary, in accordance with MDPI’s Updating Published Papers policy.
